# Energy-Based Metrics for Arthroscopic Skills Assessment

**DOI:** 10.3390/s17081808

**Published:** 2017-08-05

**Authors:** Behnaz Poursartip, Marie-Eve LeBel, Laura C. McCracken, Abelardo Escoto, Rajni V. Patel, Michael D. Naish, Ana Luisa Trejos

**Affiliations:** 1 Canadian Surgical Technologies and Advanced Robotics (CSTAR), London, ON N6A 5A5, Canada; bpoursar@uwo.ca (B.P.); mlebel4@uwo.ca (M.-E.L.); laura.c.mccracken@gmail.com (L.C.M.); abeescoto@gmail.com (A.E.); rvpatel@uwo.ca (R.V.P.); mnaish@uwo.ca (M.D.N.); 2 Department of Electrical and Computer Engineering, Western University, London, ON N6A 5B9, Canada; 3 Department of Surgery, Western University, London, ON N6A 4V2, Canada; 4 Department of Mechanical and Materials Engineering, Western University, London, ON N6A 5B9, Canada

**Keywords:** energy-based metrics, surgical skills assessment, arthroscopy, machine learning algorithms, sensorized instruments

## Abstract

Minimally invasive skills assessment methods are essential in developing efficient surgical simulators and implementing consistent skills evaluation. Although numerous methods have been investigated in the literature, there is still a need to further improve the accuracy of surgical skills assessment. Energy expenditure can be an indication of motor skills proficiency. The goals of this study are to develop objective metrics based on energy expenditure, normalize these metrics, and investigate classifying trainees using these metrics. To this end, different forms of energy consisting of mechanical energy and work were considered and their values were divided by the related value of an ideal performance to develop normalized metrics. These metrics were used as inputs for various machine learning algorithms including support vector machines (SVM) and neural networks (NNs) for classification. The accuracy of the combination of the normalized energy-based metrics with these classifiers was evaluated through a leave-one-subject-out cross-validation. The proposed method was validated using 26 subjects at two experience levels (novices and experts) in three arthroscopic tasks. The results showed that there are statistically significant differences between novices and experts for almost all of the normalized energy-based metrics. The accuracy of classification using SVM and NN methods was between 70% and 95% for the various tasks. The results show that the normalized energy-based metrics and their combination with SVM and NN classifiers are capable of providing accurate classification of trainees. The assessment method proposed in this study can enhance surgical training by providing appropriate feedback to trainees about their level of expertise and can be used in the evaluation of proficiency.

## 1. Introduction

### 1.1. Skills Assessment in Minimally Invasive Surgery

Surgical simulators are now being used for training and assessment purposes in various surgical fields including arthroscopy. The advantages of using these simulators in training programs consist of unrestricted practice time, lower cost compared to cadaver models, the opportunity for independent learning, and decreasing the risk to patients in the operating room [1]. The suitability of these simulators for training and assessment purposes not only depends on a realistic design and efficient use of the simulator, but also on the assessment method that is incorporated into the simulator to evaluate the proficiency levels of users. Objective assessment methods are essential in evaluating residents and surgeons before entering the operating room to increase safety of patients. Traditionally, skills assessment is performed by expert evaluators using global rating scales (GRS) for scoring [2]. The global rating scale for shoulder arthroscopy (GRSSA) is an example of a GRS developed for shoulder arthroscopy [3]. However, these methods are subjective and the results among different evaluators are inconsistent.

A clear definition of proficiency in minimally invasive surgery is not provided in the literature. Many studies demonstrate that a higher level of expertise is associated with a shorter task completion time [4,5]. However, quicker performance may result in reduced quality. Motion is another parameter that has been analyzed for skills assessment. Several metrics such as path length, velocity, and jerk are defined based on motion information [6,7]. The amount of force applied to the target tissue has also been considered as a representation of skill proficiency [8,9,10]. These metrics have shown high correlation with the level of expertise. However, the currently available metrics do not address all the needs and the appropriate combination of these metrics should be investigated to enhance surgical skills assessment.

Minimum energy expenditure has been identified as a feature of general motor skills [11]. Elliot et al. [12,13] demonstrated that practicing a physical task reduces energy expenditure. Analysis of energy expenditure based on instrument kinetic energy was investigated for skills assessment in [14] in the form of the integral of the acceleration vector (IAV). In our previous study, energy expenditure was introduced for laparoscopic skills assessment [15,16]. In the current study, another metric is added to the previously developed energy-based metrics, the proposed metrics are normalized, and the resulting metrics are studied for arthroscopic skills.

In order to incorporate performance metrics into surgical simulators, the criteria of expertise should be defined based on the performance of subjects with various levels of expertise. Knowing these criteria, the level of expertise of a new trainee can be determined. Machine learning algorithms are helpful in defining these criteria. As different performance metrics may demonstrate various distributions over levels of expertise, an appropriate classifying algorithm is needed for each metric or for each combination of metrics. Several machine learning algorithms have been investigated in the literature. For example, the support vector machine (SVM) is a classifying algorithm used to explore motion patterns in [17]. An accuracy of 91% was obtained in this study. Linear discriminant analysis (LDA) is another classifier used in [18,19]. In [18], LDA was used to evaluate the combination of time, force, and motion-based metrics and 100% accuracy in classifying subjects into two groups (experts and novices) was achieved. LDA was also utilized in [20] to investigate eye metrics, which were developed based on pupillary and eye movements, and provided 91.9% accuracy. The use of neural networks (NNs) was also explored in this study, resulting in 92.9% accuracy using the same eye metrics. In the current study, various methods of combining the normalized energy-based metrics using machine learning algorithms are investigated.

### 1.2. Objectives

Although energy expenditure was investigated in our previous studies for two laparoscopic tasks, its applicability in different areas of Minimally Invasive Surgery (MIS) has not been explored sufficiently. The goal of this study was to introduce and evaluate normalized energy-based metrics for basic arthroscopic tasks. Evaluating the combination of these metrics with various classifiers was also among the objectives of this study.

## 2. Methods

To accomplish the aforementioned objectives, a series of experiments were performed. In Section 2.1 the experimental protocol for data collection is explained. The normalized energy-based metrics, the classifiers that were used with these metrics, and the validation procedure are presented in Section 2.2, Section 2.3, and Section 2.4, respectively.

### 2.1. Experimental Design

**Figure 1:**
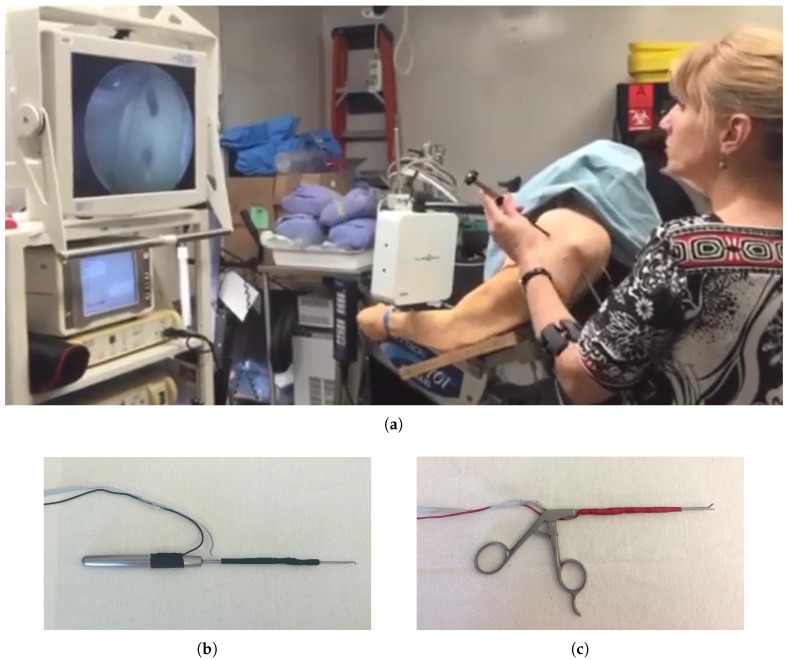
Shoulder simulator and video tower (**a**), the sensorized arthroscopic probe (**b**), and the sensorized arthroscopic grasper (**c**).

**Figure 2:**
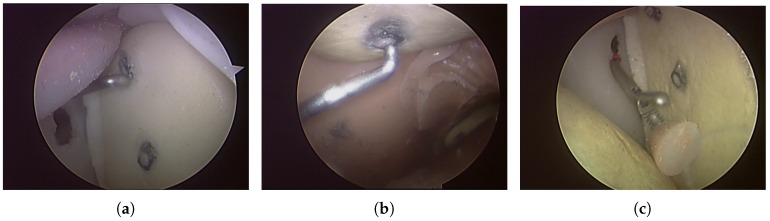
The arthroscopic tasks investigated in this study: (**a**) Task 1; (**b**) Task 2; and (**c**) Task 3.

A sensorized physical shoulder simulator was used in this study for investigating three arthroscopic tasks: two probing tasks and a grasping task. The shoulder simulator was developed at Canadian Surgical Technologies and Advanced Robotics (CSTAR), and its face and construct validity were demonstrated in [21]. The simulator and the accompanying video tower are shown in Figure 1a. The first probing task, Task 1, consisted of pressing a switch at the top and another switch in the middle of the glenoid (Figure 2a). The second probing task, Task 2, consisted of pressing a switch underneath the acromion and another switch underneath the coracoid process (Figure 2b). The switches used in the probing tasks were top-actuated switches with the operating force of 1 *N*. The successful probing of each switch was indicated by the illumination of an LED located close to the base of the simulator and was also indicated in the integrated graphical user interface of the system. The grasping task, Task 3, involved grasping and removing a loose body made of silicone from the joint capsule (Figure 2c). For all three tasks, the arthroscopic instruments were held in the left hand and the arthroscope was held in the right hand. Prior to the start of the procedure, the arthroscope was placed such that the video provided an appropriate view of the target area. The instrument was placed outside of the simulator at the opening of the appropriate portal for the task. A sensorized arthroscopic probe (Figure 1b) and a sensorized arthroscopic grasper (Figure 1c) were used for the probing and grasping tasks, respectively. These sensorized instruments were capable of measuring bending forces applied at the tip of the instrument and tracking the position of the tip of the instrument in 6 degrees of freedom (DOF). A set of four strain gauges were attached to the shaft of the instruments for force sensing, connected in a half-bridge II Wheatstone bridge. A 6 DOF position sensor (Aurora mini 6 DOF sensor, Northern Digital Inc. (NDI), Waterloo, ON, Canada), coupled to an electromagnetic position tracking system (Aurora v2, Northern Digital Inc. (NDI), Waterloo, ON, Canada), was also embedded in the shaft of each instrument and the camera [8]. The sampling frequency for both force and position data was 20 Hz. These data were then low-pass filtered with a 12-Hz cut-off frequency.

In this study, 26 participants were divided into two levels of expertise: novice (*n* = 18) and expert (*n* = 8). This grouping was performed based on each subject’s experience in arthroscopic surgery. The novice group consisted of subjects with no surgical training, orthopaedic residents, and non-orthopaedic surgeons without scoping experience. The expert group consisted of orthopaedic fellows and fellowship-trained orthopaedic surgeons. No exclusion criterion was applied for recruitment of the participants. Human Research Ethics Board approval was obtained prior to the start of the experiments from Western University Health Science Research Ethics Board (HSREB file number: 106105, Approval date 3 March 2015).

### 2.2. Metrics

The use of energy expenditure in the form of mechanical energy, including potential energy and kinetic energy, and work was proposed in our previous study for laparoscopic suturing and knot-tying tasks [15]. Work (*W*) is generated due to a force that causes a displacement. Potential energy ($E_{P}$) is due to the position of instruments in a gravitational field and kinetic energy is due to the velocity of instruments. The energy-based metrics were defined as the total work and the sum of the changes in potential energy and the sum of the changes in kinetic energy when performing a task [15]. The kinetic energy was considered due to the translational velocity of the instrument. In the current study, two forms of kinetic energy are considered: translational kinetic energy ($E_{\mathrm{TK}}$)—due to translational velocity, and rotational kinetic energy ($E_{\mathrm{RK}}$)—due to rotational velocity. The rotational kinetic-based metric is calculated according to the following formula:

(1) $$E{\;}_{\mathrm{RK}}=\int_{0}^{T}\frac{d(\omega_{x}^{2}+\omega_{y}^{2}+\omega_{z}^{2})}{dt}\;dt,$$

where *T* is task completion time, and $\omega_{x}$, $\omega_{y}$, and $\omega_{z}$ are rotational velocities about *x*, *y*, and *z* axes. If the same instrument is used to perform a task by all of the subjects, the mass of the instrument and the moment of inertia can be removed from the equations, as they would contribute the same scaling factors to the metrics of all of the subjects. In this study, the same instruments were used and the energy-based metrics did not include the mass and moment of inertia of the instruments.

Interpreting the values of the defined metrics is not possible without knowing the amount of energy expenditure corresponding to the ideal performance. In this study, an expert arthroscopist was asked to perform the tasks of this study with the same conditions as all other subjects. This expert arthroscopist had performed well over 2500 arthroscopic interventions and was also an expert with the simulator, due to her contributions to the design of the simulator and the experiment. This trial was recorded without previous practice on the same day in order to be consistent with all of the other subjects. The energy-based metrics that were calculated based on her performance were considered as the ideal metric values. Each energy-based metric was divided by the corresponding ideal value and the resulting metrics are referred to as normalized energy-based metrics ($W_{N}$, $E_{P-N}$, $E_{\mathrm{TK}-N}$, $E_{\mathrm{RK}-N}$). In other words, the normalized metrics indicate the performance of a subject relative to the ideal performance.

As the arthroscope was not sensorized with force sensors, the work-based metric was not calculated for the arthroscope. Consequently, four metrics were calculated for the instrument (($W_{N}$, $E_{P-N}$, $E_{\mathrm{TK}-N}$, $E_{\mathrm{RK}-N}$) and three metrics were calculated for the arthroscope ($E_{P-N}$, $E_{\mathrm{TK}-N}$, $E_{\mathrm{RK}-N}$).

### 2.3. Trainee Classification

In order to determine the level of expertise of trainees, a classifier should be trained with data from subjects at various levels of expertise. The classifiers should be able to accurately determine the level of expertise of subjects based on their performance metrics. The metrics used in this study were the normalized energy-based metrics as inputs to four classifiers: SVM, K-nearest neighbors (KNN), neural networks (NNs), and LDA. All of the energy-based metrics have been included in the analysis without any exclusions.

In the SVM classifier, the input data is mapped onto another feature space by a kernel function. Then the optimum hyperplane that separates the data in the mapped feature space is determined [22]. The fitcsvm function of MATLAB (Matrix Laboratory) with a linear kernel function was used to establish the SVM classifier.

KNN performs the classification based on K points that lie nearest to the test data point. The test point is assigned to the class with the highest posterior probability of class membership. This is computed as $K_{i}/K$, where $K_{i}$ is the number of points of Class *i* that lie nearest to the test point. As K increases the borders of each class become smoother, and as it decreases fine variations in each class can be determined. The choice of a large K reduces sensitivity to noise [22]; however, due to the small sample size of the current study, the choice of a large K was not possible. Considering a maximum of 6 valid trials for experts (as described in Section 3), K was assigned a value of 3 in this study.

NNs were also investigated through the neural network toolbox of MATLAB. As suggested in the literature, the maximum number of hidden layer nodes should be *N*/*d*, where *N* is the length of the training data and *d* is the number of input nodes [23]. For all three tasks of this study, the network structure consisted of 3 input nodes when the energy-based metrics of the arthroscope were considered, 4 input nodes when the energy-based metrics of the instrument were considered, and 7 input nodes when the energy-based metrics of both of the instrument and the arthroscope were considered. In addition, one hidden layer with 3 nodes and 1 output node were specified in the network structure. This structure reduces computational cost and the possibility of overfitting. The training data were divided into two subsets: 70% for network training and 30% for training validation. The optimization of the weights and bias was performed by the Levenberg–Marquardt backpropagation algorithm. The target matrix was set to 1 for novices and 2 for experts. The output of the NN model was then rounded to assign the test data point to its corresponding group. In the LDA algorithm, the multi-dimensional feature matrix is projected into one dimension by multiplying the feature matrix by a weight vector. This weight vector is determined in a manner that maximizes the separation of class means and minimizes interclass variance [22]. The fitcdiscr function of MATLAB was used to implement the LDA classifier.

### 2.4. Validation

#### 2.4.1. Leave-One-Subject-Out Cross-Validation

Validation of the proposed metrics and the combination of these metrics with the above-mentioned classifiers was performed through a leave-one-subject-out (LOSO) cross-validation technique. In this technique, the data is partitioned into two sets: a *test* set, consisting of one subject, and a *training* set, consisting of all subjects except the test subject. The validation procedure is repeated with different test subjects until all the subjects have been in the test group once [17,18,22,24]. The level of expertise of the test subject is determined in the validation procedure, assuming that his/her level of expertise is unknown. The determined level of expertise is then compared to the level of expertise of subjects based on their experience in arthroscopic surgery.

The performance of these classifiers in combination with the normalized energy-based metrics was quantified through four measures: accuracy—ratio of the total number of correct identifications to the total number of subjects, precision—ratio of the number of experts classified as expert to the number of subjects classified as expert, recall—ratio of the number of expert subjects classified as experts to the total number of experts, and F1 score, which is defined as:

(2) $$F1=2\times \frac{\mathrm{precision}\times \mathrm{recall}}{\mathrm{precision}+\mathrm{recall}}.$$

Mistakenly classifying experts as novices indicates that they require more practice, however, wrongly classifying novices as experts can result in safety issues for patients. Consequently, it is very important to investigate the ability of the assessment method to correctly classify experts, which can be evaluated by precision and recall measures. The F1 score is the harmonic mean of precision and recall, which is the appropriate method of calculating the average of parameters that are represented as percentages. In other words, the F1 score demonstrates the balance between precision and recall [25,26].

The performance of the energy-based metrics is also compared to the combination of task completion time, path length, and maximum bending force. This combination is evaluated using the LOSO cross validation for all of the classifiers that are investigated in this study.

#### 2.4.2. Computation Time

In order to compare the computation times of the classifiers, the running time for training the classifiers and testing of all the subjects in the cross-validation was measured. The stopwatch timer of MATLAB was employed for the three tasks of this study and the mean and standard deviation values were calculated. Statistical analysis was also performed to investigate the difference between the classifiers in terms of the running time. All computations were implemented on a PC running Windows 7 with a 3.40 GHz Intel(R) Core(TM) i7-3770 CPU and 8 GB RAM.

## 3. Results

The recorded data were explored to remove any erroneous data from the analysis. The data sets that contained significant interruptions in the recording were excluded from the study. These interruptions could happen due to limited range of position tracking, sensitivity of the position tracking system to ferromagnetic metal, or a disconnection in the force sensing circuit. Therefore, the number of subjects for which valid data were recorded varied in different tasks. Similarly, for analysis of both hands together, the subjects whose data from either the instrument or the arthroscope was not valid were excluded. Table 1 shows the number of subjects with valid data from the instrument, the arthroscope, and both the instrument and arthroscope.

**Figure 3:**
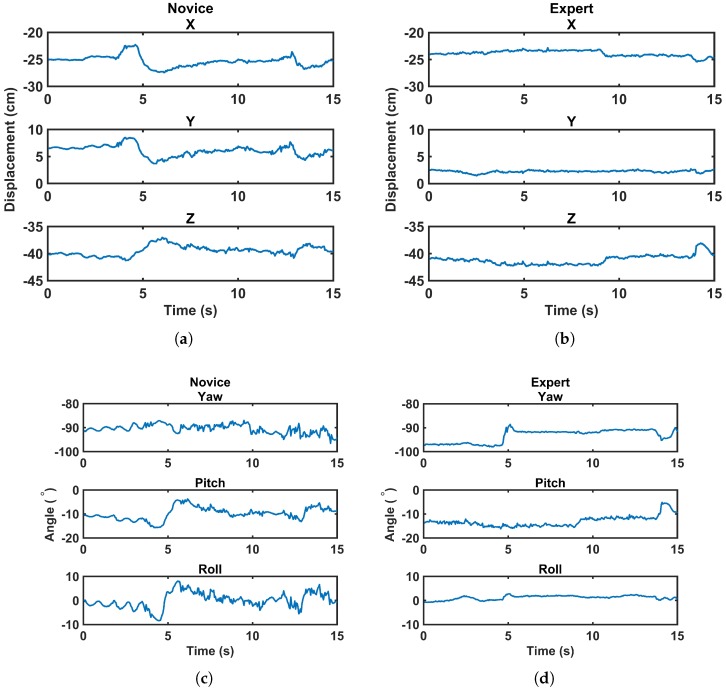
The arthroscope’s tip displacement (**a**,**b**), and angle (**c**,**d**) for a random novice and expert subject over the same time duration.

The experimental design of this study required holding the arthroscope in an appropriate position at the beginning of the task. In Task 1, both switches were clearly visible in front of the camera at the beginning. However, subjects were allowed to move the arthroscope as required, e.g., to zoom in on the switch or find the instrument tip. In Task 2, the switch underneath the acromion was clearly visible at the beginning, but to have an appropriate view of the switch underneath the coracoid process, subjects needed to navigate around the coracoid. In Task 3, the arthroscope was located in a position that showed the loose body, but it could be re-positioned by the subject as needed. Although the main part of the task was supposed to be completed by manipulating the instrument, the use of the arthroscope was affected by the expertise of the subjects as well. Figure 3 provides a comparison of the changes in the displacement and angle of the arthroscope in 6 DOF between a random novice and a random expert during a 15-s time frame. In this figure, the same vertical limits are applied for both the novice and expert subjects to provide a clear comparison, i.e., 10 cm for displacement and 20${}^{\circ }$ for angle. More fluctuations and changes in position and angle of the arthroscope can be seen for the novice subject compared to the expert one. These fluctuations result in a higher energy expenditure by the novice subjects.

### 3.1. Energy-Based Metrics and Normalized Energy-Based Metrics

**Figure 4:**
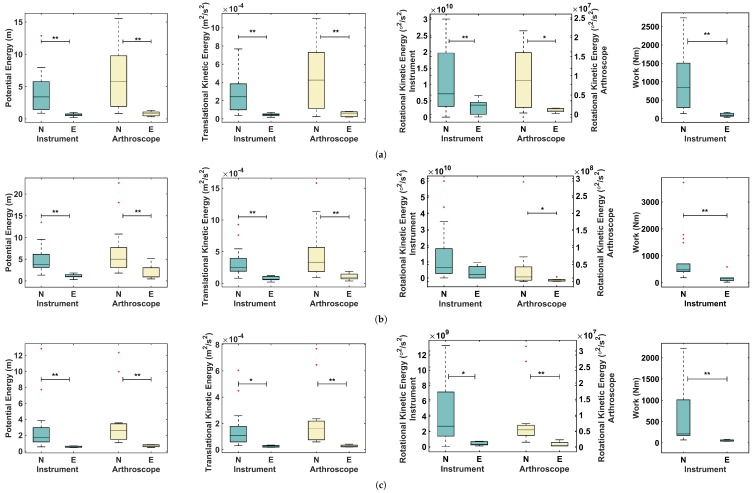
Energy-based metrics for the instrument (left) and arthroscope (right). (**a**) Task 1, (**b**) Task 2, and (**c**) Task 3. In this figure, ** indicates a statistically significant difference with *p* value less than 0.01 and * indicates a statistically significant difference with *p* value less than 0.05.

The valid data were used to calculate energy-based metrics for the left hand (holding the proper instrument for the task) and the right hand (holding the arthroscope). As can be seen in Figure 4, the amount of energy expenditure for the experts was considerably lower than that for the novices. As seen in this figure, Task 2 required higher levels of energy than Tasks 1 and 3. This is due to the position of the switches, which required more effort, even by experts. Tasks 1 and 3 required similar ranges of energy in terms of potential energy, translational kinetic energy, and work. However, the required amount of rotational energy for Task 3 was considerably less than the corresponding value for Task 1. The Probing tasks required manipulation of the probe in certain angles to successfully press the switches, which was not required in Task 3. Regarding the outliers in Figure 4, the videos of subjects who were recognized as outliers were inspected to find any external reason that might affect their performance. As these outlier points were not related to a reasonable cause, they were included in the analysis.

The normality of the results for each metric was analyzed using the Shapiro–Wilk test through the Statistical Package for the Social Sciences, Version 24 (SPSS, Chicago, IL, USA). The normality test was rejected for some of the energy-based metrics in different tasks. The metrics with a normal distribution were analyzed using the Independent-Sample *t* test and the metrics with non-normal distribution were analyzed using the Mann–Whitney U test of SPSS. The statistical analysis showed a significant difference between the two levels of expertise for all the normalized energy-based metrics except rotational kinetic energy of the instrument for Task 2. These metrics were then normalized with respect to the corresponding values of the ideal performance of each task as was explained in Section 2.2. The mean and standard deviation of the resulting metrics, the normalized energy-based metrics, are shown in Table 2. Statistical analysis was also performed on these metrics using the Independent-Sample *t* test or the Mann–Whitney U test depending on whether the data presented a normal or non-normal distribution. The metrics with a normal distribution are marked by an asterisk in the *p* value columns of Table 2. For most of the normalized energy-based metrics, the mean values of the expert group were close to 1 and there was a significant difference between the expert and the novice groups. The small variance among the expert group demonstrates the similarity of the performance of the expert subjects to the ideal performance. The only metric that had a mean value considerably higher than 1 was the rotational kinetic energy for Task 1. This can be due to the unfamiliarity of the subjects with the appropriate angle of holding the instrument when pressing the switches. This metric decreases significantly in Task 2.

### 3.2. Validation

**Figure 5:**
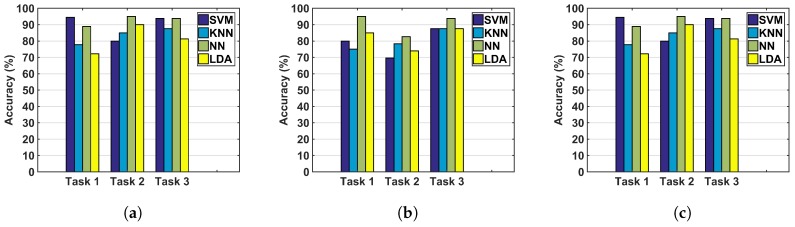
Accuracy using (**a**) only the instrument’s metrics; (**b**) using only the arthroscope’s metrics; and (**c**) using metrics of both the instrument and the arthroscope. SVM: support vector machine; KNN: K-nearest neighbors; NN: neural network; LDA: linear discriminant analysis.

The accuracy of classification using the normalized metrics and the investigated classifiers are shown in Figure 5. Overall, considering the metrics of the arthroscope as the only inputs to the classifiers provides lower accuracy levels than incorporating the metrics of the instruments in the classification. In addition, the NN method demonstrated higher accuracy levels compared to the other classifiers. Accuracy, precision, recall, and F1 score, for using the normalized energy-based metrics of both hands, including the metrics of the instruments and the arthroscope together, are shown in Table 3. Although the results were superior for the instrument only, the inclusion of both hands was considered to be the broader use of the metrics and the corresponding results are reported to allow for comparison of the different classifiers. The NN method provides the highest accuracy for nearly all of the tasks and different input metrics. NNs also demonstrate precision levels higher than 75%.

Temporal, motion-based and force-based metrics were calculated in a previous study for the same data set [27]. The results of [27] showed statistically significant differences between the experts and novices for most of the investigated metrics. The performance of the classifiers in conjunction with task time, path length for both the instrument and the arthroscope, and maximum bending force were evaluated and the results are presented in Table 4. As can be seen, the results that were obtained using the normalized energy-based metrics provide superior accuracy, precision, and recall in a larger number of conditions of using different classifiers and tasks. However, for some of the conditions, such as using NN for Tasks 2 and 3, both the energy-based metrics and the non-energy metrics provide similar accuracy levels.

The running times were also measured for different tasks and the mean and standard deviations are represented in Table 5 for various classifiers. As can be seen, NNs require the maximum running time among the four classifiers investigated in this study. The difference between these running times was investigated using Kruskal–Wallis test, followed by post-hoc tests. The results of statistical analysis showed that the running time of NN is significantly different from that of the KNN, SVM, and LDA with the following *p* values, respectively: <0.001, 0.001, and 0.044. In addition, the running time of KNN and LDA were also significantly different (*p* = 0.001).

## 4. Discussion

The goal of this study was to develop new metrics for arthroscopic skills assessment and evaluate the use of these metrics with different classifiers to determine a subject’s level of expertise. The results of this study are discussed in detail in the following sections.

### 4.1. Normalized Energy-Based Metrics

All energy-based metrics showed higher levels of energy expenditure for novices compared to experts. This is due to a larger number of movements of the instrument or the arthroscope and higher levels of applied forces that were unnecessary for completion of the task. These unnecessary forces and movements can be due to lack of appropriate control over the instrument or the arthroscope. The tasks we studied were designed to focus on the performance of the instrument. However, it was noticed that there were significant differences in manipulating the arthroscope between the experts and novices. The unnecessary arthroscope movements may have been generated as a result of motor overflow, which can occur in effortful actions [28,29]. It was also observed in [30] that an unsuccessful navigation in cadaver models using an arthroscope generates large number of fluctuations in the applied force. The arthroscopic tasks studied here were comprehended as complicated motor activities for many of the novices. The statistical significant difference between novices and experts and the approximately similar accuracy levels that various classifiers provided for each task support the presence of a strong relationship between the normalized energy-based metrics and level of expertise. The comparison between the energy-based metrics and the combination of time, path length, and maximum force showed that higher accuracy levels can be achieved for all three tasks studied using energy-based metrics in conjunction with some of the classifiers such as SVM.

### 4.2. Instrument, Arthroscope, or Both?

A maximum accuracy of 95% was obtained for all three input conditions, Figure 5. However, the overall accuracy levels for different tasks were lower when the arthroscope’s metrics were the only inputs to the classifiers. Regarding the arthroscope’s metrics, it should be considered that these metrics were developed based on the motion parameters only and the work-based metric was not calculated. This indicates the importance of measuring force for surgical skills assessment, which is in accordance with the results found in [18,31]. The inferior results of skills assessment based on the arthroscope’s metrics can be due to the absence of a work-based metric in the assessment or because of the secondary role of the arthroscope in performing the tasks. However, for other tasks that require further navigation of the arthroscope, more accurate identification might be obtained by incorporating the arthroscope’s metrics.

### 4.3. Classifiers

The classifiers investigated in this study are among the machine learning algorithms that do not require heavy computations. These classifiers provided approximately similar results. However, the KNN and LDA have demonstrated the minimum accuracy and precision among the classifiers used. The LDA reduces the dimension of the input data and in this procedure tries to maximize the distance between the mean values of the two groups. However, the difference between the mean values of the two groups is not usually the best criterion of discrimination. Since normality is among the assumptions of the LDA, another reason for the low accuracy of this classifier may be the non-normal distribution of some of the normalized energy-based metrics. The KNN classifiers do not require a particular distribution of the samples, but have shortcomings such as sensitivity to the local structure of the data and the curse of dimensionality. In addition, the performance of KNN is affected by the value of K, which in our study was limited due to the limited number of experts.

SVM and NNs provided promising results. The range of accuracy of NNs was 89–95%. In this study, a simple configuration was considered for the NN to prevent overfitting. This method is robust to an increase in the number of inputs and is also capable of learning non-linear relationships. However, a dependency on the initial conditions and a large computational burden can be cited as disadvantages of this method. SVM provides a unique solution for classification and offers a reasonable computational time. This method provides the highest accuracy levels (95%) but when considering the arthroscope’s metrics for Task 2, SVM did not demonstrate a high accuracy.

The results of our study are comparable to the results of previous studies in surgical skills assessment. According to our results, the groups of novices and experts can be discriminated with 95% accuracy, which is slightly higher than the results reported in [17,20] (92%) and is slightly lower than the results of [18] (100%). However, it should be noted that these results also depend on the tasks studied, the diversity of subjects, and data recording methods. The results of our method, which are also close to the accuracy level of previous studies, demonstrate the high potential of the proposed metrics and classifiers for surgical skills assessment.

### 4.4. Tasks

In this study, two probing tasks (Task 1 and Task 2) were investigated in different shoulder locations. The two non-significant differences between novices and experts were found for the normalized rotational kinetic energy for Task 2. The difficult posture required to press the switches in this task increased the complexity of the task, even for some of the expert subjects. This task might be valuable for studies that also investigate intermediate levels of expertise. Task 1 and Task 3 demonstrated suitable levels of difficulty for distinguishing the two levels of expertise. However, performing Task 3 in a wet environment—closer to a real surgical condition— can possibly increase the difficulty of this task by impacting the degree of visibility of the anatomical structures.

To summarize, the energy-based metrics were analyzed for the first time for arthroscopic tasks. In addition, a new energy-based metric, rotational kinetic energy, was proposed and evaluated. In this study, the role of the arthroscope was secondary relative to the role of the other instrument in completing the tasks. However, it was shown that even for the arthroscope, there was a significant difference between experts and novices in terms of the energy-based metrics. The normalization of the metrics provided additional information about variation in performance in the novice and expert groups. Furthermore, various machine learning algorithms were evaluated in conjunction with the normalized metrics to establish the appropriate combination of the proposed metrics, and their performances were evaluated by implementing various measures. Although this study uses some of the energy-based metrics that were introduced in our earlier study, several new aspects of their use have been investigated for the first time and have been modified to improve the quality of skills assessment. In addition to the above novelties and in comparison with other studies in the area of surgical skills assessment, this study evaluates various machine learning algorithms for the normalized energy-based metrics and for arthroscopic tasks.

## 5. Conclusions and Future Work

This study proposed novel performance metrics based on normalized mechanical energy and work. The incorporation of these metrics for arthroscopic skills assessment was studied. For this purpose, various machine learning classifiers were investigated, among which support vector machines (SVM) and neural networks (NNs) demonstrated high discrimination capabilities. The validation results showed that these metrics are capable of differentiating between novices and experts with 95% accuracy. It was also demonstrated that the work-based metrics can enhance the accuracy of classification. Consequently, it is recommended that force sensing is incorporated into data recording system to establish a more accurate assessment method. Overall, our results show that normalized energy-based metrics can enhance arthroscopic skills assessment. The normalization of the metrics using ideal performance metrics allows trainees to compare their performance with the ideal performance.

One of the future works of this study is to record further performance data for the arthroscopic tasks. Larger numbers of samples would provide more comprehensive models of performance at each level of expertise. In particular, more data related to expert performance can be used to further refine the criteria of expertise. Investigating the use of these metrics for finer classification of the levels of expertise, including intermediate levels, is another future direction of this study. In addition, the appropriate form of using the energy-based metrics for providing feedback and the methods for presenting this data to trainees need to be explored as part of future work.

## Figures and Tables

**Table 1:** Number of subjects with valid data from the instrument, the arthroscope, and both the instrument and the arthroscope for the three studied tasks.

Task	Instrument			Arthroscope			Instrument and Athroscope		
	Novices	Experts	Total	Novices	Experts	Total	Novices	Experts	Total
1	16	4	20	15	5	20	14	4	18
2	18	5	23	17	6	23	15	5	20
3	16	4	20	12	4	16	12	4	16

**Table 2:** The mean and standard deviation of the normalized energy-based metrics for the novice and expert groups and the corresponding *p* values. The statistically significant *p* values are shown in bold. $E_{P-N}$, $E_{\mathrm{TK}-N}$, $E_{\mathrm{RK}-N}$, and $W_{N}$ stand for the normalized potential energy, normalized translational kinetic energy, normalized rotational kinetic energy, and normalized work, respectively. All of the metrics, except $E_{\mathrm{RK}-N}$ of the instrument and the arthroscope for Task 2, which are indicated with an asterisk (*), demonstrated statistically significant differences between novices and experts. The metrics with a normal distribution are marked with † in the *p* value column.

			Task 1		Task 2		Task 3	
	Metric	Level	Mean ± SD	*p* value	Mean ± SD	*p* value	Mean ± SD	*p* value
Instrument	$E_{P-N}$	Novice	5.87 ± 4.47	**0.001**	5.15 ± 3.26	**<0.001**	5.22 ± 5.55	**0.002**
		Expert	0.88 ± 0.44		1.18 ± 0.60		1.10 ± 0.17	
	$E_{\mathrm{TK}-N}$	Novice	6.24 ± 4.65	**<0.001 ^†^**	5.92 ± 4.12	**0.001**	6.92 ± 6.98	**0.022**
		Expert	1.07 ± 0.51		1.37 ± 0.74		1.84 ± 1.29	
	$E_{\mathrm{RK}-N}$	Novice	145.35 ± 127.06	**0.014 ^†^**	8.05 ± 10.15	0.199 *	5.10 ± 3.33	**0.003 ^†^**
		Expert	50.56 ± 29.24		2.41 ± 2.65		1.95 ± 0.78	
	$W_{N}$	Novice	29.43 ± 22.71	**<0.001 ^†^**	9.92 ± 10.16	**0.007**	7.64 ± 7.89	**0.001**
		Expert	3.49 ± 1.32		2.68 ± 2.52		0.53 ± 0.21	
Arthroscope	$E_{P-N}$	Novice	11.89 ± 9.35	**0.001 ^†^**	7.07 ± 6.10	**0.024**	8.34 ± 8.02	**0.001**
		Expert	1.59 ± 0.79		2.34 ± 1.93		2.23 ± 1.56	
	$E_{\mathrm{TK}-N}$	Novice	10.72 ± 8.74	**0.011**	6.44 ± 5.47	**0.002**	8.38 ± 8.56	**0.001**
		Expert	1.40 ± 0.77		1.58 ± 0.79		1.84 ± 1.88	
	$E_{\mathrm{RK}-N}$	Novice	17.23 ± 23.34	**0.042**	10.95 ± 19.03	0.062 *	10.46 ± 11.53	**0.002**
		Expert	1.56 ± 0.79		1.44 ± 1.36		1.09 ± 1.10	

**Table 3:** Accuracy, precision, recall, and F1 score as percentages when the normalized energy-based metrics of both the instrument and the arthroscope are used as inputs of the classifiers.

Task	Classifier	Accuracy	Precision	Recall	F1 score
1	SVM	94.44	80.00	100.00	88.89
	KNN	77.78	50.00	75.00	60.0
	NN	88.89	75.00	75.00	75.00
	LDA	72.22	42.85	75.00	54.55
2	SVM	80.00	57.14	80.00	66.67
	KNN	85.00	75.00	60.00	66.67
	NN	95.00	100.00	80.00	88.89
	LDA	90.00	80.00	80.00	80.00
3	SVM	93.75	80.00	100.00	88.89
	KNN	87.50	66.67	100.00	80.00
	NN	93.75	80.00	100.00	88.89
	LDA	81.25	60.00	75.00	66.67

**Table 4:** Accuracy, precision, recall, and F1 score as percentages when task time, path length of both the instrument and the arthroscope, and maximum bending force of the instrument are used as inputs to the classifiers.

Task	Classifier	Accuracy	Precision	Recall	F1 score
1	SVM	66.67	25.00	25.00	25.00
	KNN	61.11	0.00	0.00	0.00
	NN	83.33	66.67	50.00	57.14
	LDA	88.89	75.00	75.00	75.00
2	SVM	80.00	66.67	40.00	50.00
	KNN	75.00	50.00	20.00	28.57
	NN	95.00	100.00	80.00	88.89
	LDA	80.00	100.00	20.00	33.33
3	SVM	81.25	60.00	75.00	66.67
	KNN	87.50	75.00	75.00	75.00
	NN	93.75	100.00	75.00	85.71
	LDA	87.50	100.00	50.00	66.67

**Table 5:** Mean and standard deviation of the running time for different classifiers.

Classifier	SVM	KNN	NN	LDA
Running time (s) (Mean ± SD)	0.969 ± 0.028	0.866 ± 0.039	3.290 ± 0.452	1.015 ± 0.033

## References

[1] Roberts P.G., Guyver P., Baldwin M., Akhtar K., Alvand A., Price A.J., Rees J.L. (2016). Validation of the updated ArthroS simulator: Face and construct validity of a passive haptic virtual reality simulator with novel performance metrics. Knee Surg. Sports Traumatol. Arthrosc..

[2] Reiley C.E., Lin H.C., Yuh D.D., Hager G.D. (2011). Review of methods for objective surgical skill evaluation. Surg. Endosc..

[3] Hoyle A.C., Whelton C., Umaar R., Funk L. (2012). Validation of a global rating scale for shoulder arthroscopy: A pilot study. Shoulder Elb..

[4] Tashiro Y., Miura H., Nakanishi Y., Okazaki K., Iwamoto Y. (2009). Evaluation of skills in arthroscopic training based on trajectory and force data. Clin. Orthop. Relat. Res..

[5] Martin K.D., Belmont P.J., Schoenfeld A.J., Todd M., Cameron K.L., Owens B.D. (2011). Arthroscopic basic task performance in shoulder simulator model correlates with similar task performance in cadavers. J. Bone Joint Surg..

[6] Escoto A., Le Ber F., Trejos A.L., Naish M.D., Patel R.V., LeBel M.E. A knee arthroscopy simulator: Design and validation. Proceedings of the 35th Annual International Conference of the IEEE Engineering in Medicine and Biology Society (EMBC).

[7] Oropesa I., Sánchez-González P., Lamata P., Chmarra M.K., Pagador J.B., Sánchez-Margallo J.A., Sánchez-Margallo F.M., Gómez E.J. (2011). Methods and tools for objective assessment of psychomotor skills in laparoscopic surgery. J. Surg. Res..

[8] Escoto A., Trejos A.L., Naish M.D., Patel R.V., LeBel M.E. (2012). Force sensing-based simulator for arthroscopic skills assessment in orthopaedic knee surgery. Medicine Meets Virtual Reality.

[9] Puangmali P., Althoefer K., Seneviratne L.D., Murphy D., Dasgupta P. (2008). State-of-the-art in force and tactile sensing for minimally invasive surgery. IEEE Sens. J..

[10] Cutler N., Balicki M., Finkelstein M., Wang J., Gehlbach P., McGready J., Iordachita I., Taylor R., Handa J.T. (2013). Auditory force feedback substitution improves surgical precision during simulated ophthalmic surgery. Investig. Ophthalmol. Vis. Sci..

[11] Guthrie E.R. (1952). The Psychology of Learning.

[12] Elliott D., Grierson L.E., Hayes S.J., Lyons J. (2011). Action representations in perception, motor control and learning: implications for medical education. Med. Educ..

[13] Elliott D., Hansen S., Mendoza J., Tremblay L. (2004). Learning to optimize speed, accuracy, and energy expenditure: a framework for understanding speed-accuracy relations in goal-directed aiming. J. Mot. Behav..

[14] Cavallo F., Sinigaglia S., Megali G., Pietrabissa A., Dario P., Mosca F., Cuschieri A. (2014). Biomechanics–machine learning system for surgical gesture analysis and development of technologies for minimal access surgery. Surg. Innov..

[15] Poursartip B., LeBel M.E., Patel R.V., Naish M.D., Trejos A.L. Energy-based metrics for laparoscopic skills assessment. Proceedings of the 38th Annual International Conference of the IEEE Engineering in Medicine and Biology Society (EMBC).

[16] Poursartip B., LeBel M.E., Patel R.V., Naish M.D., Trejos A.L. Analysis of energy-based metrics for laparoscopic skills assessment. Accepted for publication in the IEEE Transactions on Biomedical Engineering 2017.

[17] Ahmidi N., Poddar P., Jones J.D., Vedula S.S., Ishii L., Hager G.D., Ishii M. (2015). Automated objective surgical skill assessment in the operating room from unstructured tool motion in septoplasty. Int. J. Comput. Assist. Radiol. Surg..

[18] Horeman T., Dankelman J., Jansen F.W., van den Dobbelsteen J.J. (2014). Assessment of laparoscopic skills based on force and motion parameters. IEEE Trans. Biomed. Eng..

[19] Chmarra M.K., Klein S., de Winter J.C., Jansen F.W., Dankelman J. (2010). Objective classification of residents based on their psychomotor laparoscopic skills. Surg. Endosc..

[20] Richstone L., Schwartz M.J., Seideman C., Cadeddu J., Marshall S., Kavoussi L.R. (2010). Eye metrics as an objective assessment of surgical skill. Ann. Surg..

[21] McCracken L.C. (2015). Development of a Physical Shoulder Simulator for the Training of Basic Arthroscopic Skills. Master’s Thesis.

[22] Bishop C. (2007). Pattern Recognition and Machine Learning (Information Science and Statistics).

[23] Xu S., Chen L. A novel approach for determining the optimal number of hidden layer neurons for FNN’s and its application in data mining. Proceedings of the 5th International Conference on Information Technology and Applications (ICITA).

[24] Reiley C.E., Hager G.D. (2009). Decomposition of robotic surgical tasks: An analysis of subtasks and their correlation to skill. M2CAI Workshop.

[25] Despinoy F., Bouget D., Forestier G., Penet C., Zemiti N., Poignet P., Jannin P. (2016). Unsupervised trajectory segmentation for surgical gesture recognition in robotic training. IEEE Trans. Biomed. Eng..

[26] Kumar R., Jog A., Malpani A., Vagvolgyi B., Yuh D., Nguyen H., Hager G., Chen C.C.G. (2012). Assessing system operation skills in robotic surgery trainees. Int. J. Med. Robot. Comput. Assist. Surg..

[27] Poursartip B., McCracken L., Escoto A., Patel R., LeBel M., Trejos A., Naish M. Development and evaluation of a sensorized shoulder simulator. Proceedings of the 30th Canadian Conference of the IEEE Electrical and Computer Engineering (CCECE).

[28] Bodwell J.A., Mahurin R.K., Waddle S., Price R., Cramer S.C. (2003). Age and features of movement influence motor overflow. J. Am. Geriatr. Soc..

[29] Addamo P.K., Farrow M., Bradshaw J.L., Georgiou-Karistianis N. (2011). Relative or absolute? Implications and consequences of the measures adopted to investigate motor overflow. J. Mot. Behav..

[30] Horeman T., Tuijthof G., Wulms P., Kerkhoffs G., Gerards R., Karahan M. (2016). A Force Measurement System for Training of Arthroscopic Tissue Manipulation Skills on Cadaveric Specimen. J. Med. Devices.

[31] Trejos A.L., Patel R.V., Malthaner R.A., Schlachta C.M. (2014). Development of force-based metrics for skills assessment in minimally invasive surgery. Surg. Endosc..

